# Genetic contributions to the stability and satisfaction in Sexual Relationships

**DOI:** 10.1016/j.gmg.2025.100043

**Published:** 2025-02-20

**Authors:** Kirolos Eskandar

**Affiliations:** Helwan University, Faculty of Medicine and Surgery, Egypt

**Keywords:** Genetics, Relationship stability, Relationship satisfaction, Personality traits, Attachment styles, Emotional regulation, Hormonal influences, Sexual compatibility, Communication styles, Gene-environment

## Abstract

The stability and satisfaction of sexual relationships are critical determinants of individual well-being and societal cohesion. While much is known about the psychological and social factors influencing these outcomes, the genetic underpinnings remain an emerging field of inquiry. This literature review synthesizes findings from 42 peer-reviewed studies published between 2003 and 2023, exploring the genetic contributions to relationship stability and satisfaction. Key findings indicate that neuroticism, with an estimated heritability of ∼40 %, is a strong predictor of relationship instability, while agreeableness and extraversion are associated with greater relationship satisfaction. The review examines the genetic foundations of personality traits, attachment styles, emotional regulation, hormonal influences, sexual compatibility, communication styles, and mental health predispositions. Additionally, it highlights the interplay between genetic and environmental factors, presenting case studies and empirical evidence that elucidate the complex interactions at play. Ethical considerations and future research directions are discussed to provide a comprehensive understanding of how genetics can shape successful sexual relationships. By bridging the gap between genetic research and relationship science, this review offers data-driven insights to guide future investigations in this interdisciplinary domain.

## Introduction

The stability and satisfaction of sexual relationships are foundational to individual well-being and societal harmony. Stable and satisfying relationships are associated with numerous positive outcomes, including enhanced mental health, greater life satisfaction, and increased longevity. Conversely, relationship instability and dissatisfaction can lead to significant psychological distress, manifesting in anxiety, depression, and decreased overall quality of life [1]. The intricate dynamics of relationships are influenced by a myriad of factors, ranging from social and environmental influences to individual psychological traits. Among these, genetic factors are gaining increasing recognition for their role in shaping relationship outcomes [2].

Recent advancements in behavioral genetics have illuminated the substantial influence of genetic factors on various aspects of human behavior, including interpersonal relationships. Genetic predispositions can affect personality traits, emotional regulation, and even specific behavioral tendencies that are pivotal in forming and maintaining relationships. For instance, studies have demonstrated that certain genetic variations, such as those affecting the serotonin transporter gene (SLC6A4), are associated with traits like neuroticism and agreeableness, which in turn influence relationship satisfaction and stability [3]. These genetic factors can predispose individuals to particular patterns of behavior and emotional responses that either foster or hinder relationship success.

Moreover, the field of attachment theory, which explores how early life experiences with caregivers shape one’s approach to relationships, has been augmented by genetic research. Genetic predispositions can influence attachment styles, affecting how individuals form bonds and respond to intimacy and conflict within relationships [4]. For example, genetic variations in the oxytocin receptor gene (OXTR) have been linked to differences in social bonding and attachment behaviors, providing a biological basis for these critical relational patterns.

To make this topic accessible to an interdisciplinary audience, it is important to define key genetic terms. Heritability refers to the proportion of variance in a trait attributable to genetic factors within a given population. Single nucleotide polymorphisms (SNPs) are variations in a single DNA building block, which can influence traits such as emotional regulation. Polygenic risk scores (PRS) aggregate the effects of multiple genetic variants to estimate an individual’s predisposition to certain behaviors, including those affecting relationship satisfaction.

In addition to personality and attachment styles, emotional regulation is another key area where genetics plays a significant role. Effective emotional regulation is crucial for maintaining relationship harmony, especially during conflicts. Genetic studies have identified specific alleles that influence the functioning of neurotransmitters like serotonin and dopamine, which are integral to emotional regulation [5]. Individuals with genetic variations that promote better emotional control are likely to experience greater relationship satisfaction and stability.

Hormonal influences also intersect with genetic factors to impact relationships. For instance, variations in genes related to the production and reception of hormones such as oxytocin and vasopressin have been shown to affect social bonding and partner interactions [6]. These hormonal pathways are critical in fostering intimacy and trust, essential components of a successful relationship.

Sexual compatibility, a crucial aspect of relationship satisfaction, may also have a genetic component. Research suggests that genetic compatibility, particularly in the context of the major histocompatibility complex (MHC), can influence sexual attraction and partner selection, thereby impacting relationship stability [7]. Genetic compatibility might contribute to the biological basis for the often subconscious drive toward certain partners, thus playing a role in the success of the relationship.

Communication styles, which are pivotal for resolving conflicts and expressing needs within a relationship, are another domain influenced by genetic factors. Genetic research has shown that communication patterns can be partially inherited, affecting how effectively partners navigate relational challenges [8]. Understanding these genetic influences provides a deeper insight into the biological underpinnings of relationship dynamics.

Finally, mental health, which is significantly influenced by genetic predispositions, plays a critical role in relationship stability and satisfaction. Genetic vulnerabilities to mental health disorders such as depression and anxiety can strain relationships, making it essential to consider these factors when examining relationship outcomes [9]. The interplay between genetic predispositions and environmental triggers highlights the complexity of relationships and the multifaceted nature of their success or failure. Despite growing evidence of genetic influences on relationships, the role of epigenetics—modifications in gene expression due to environmental factors—remains underexplored and represents a key research gap that future studies should address.

### Methodology

This systematic literature review was conducted to synthesize existing research on the genetic contributions to stability and satisfaction in sexual relationships. The following methodological framework was adhered to, ensuring a comprehensive and systematic approach to data collection, evaluation, and synthesis.

### Search strategy

A thorough search was performed across multiple reputable databases, including PubMed, Scopus, Web of Science, and Google Scholar. These databases were selected for their extensive coverage of genetic, psychological, and social science research. The search strategy was developed using a combination of key terms and Boolean operators to ensure comprehensive retrieval of relevant literature. Keywords included "Genetics," "Relationship Stability," "Relationship Satisfaction," "Personality Traits," "Attachment Styles," "Emotional Regulation," "Hormonal Influences," "Sexual Compatibility," "Communication Styles," and "Gene-Environment Interactions.".

Boolean operators such as AND, OR, and NOT were employed to refine search results and avoid redundant or irrelevant articles. For instance, "Genetics AND Relationship Stability" and "Hormonal Influences OR Attachment Styles" were used to target specific intersections of the topic. Searches were restricted to English-language publications to maintain consistency and comprehensibility.

### Inclusion and exclusion criteria

Studies were selected based on the following inclusion criteria:

• Language: Articles published in English.
• Population Focus: Studies focusing specifically on sexual relationships.
• Relevance: Research reporting on genetic contributions to stability and satisfaction in relationships.
• Publication Type: Peer-reviewed journal articles, systematic reviews, and meta-analyses.
• Time Frame: Studies published within the last 20 years (2003-2023), ensuring the inclusion of contemporary research.

### Exclusion criteria included

• Non-peer-reviewed articles, such as opinion pieces and editorials.
• Studies focusing solely on non-human populations.
• Research that did not explicitly discuss genetic contributions to relationship outcomes.
• Duplicates from different databases.

### Screening process

An initial search yielded 113 articles. The articles were screened in a two-step process:

• Title and Abstract Screening: Articles were reviewed for relevance based on their titles and abstracts. Studies that clearly met the inclusion criteria were retained.
• Full-Text Review: The remaining articles underwent a detailed review of their full texts to confirm their alignment with the study’s objectives and inclusion criteria.

After removing duplicates and irrelevant studies, 42 unique articles were selected for inclusion in this review.

### Quality assessment

To ensure the reliability and validity of the included studies, a quality assessment was conducted using standardized tools, such as the Critical Appraisal Skills Programme (CASP) checklist. Each study was evaluated on its methodology, sample size, data analysis, and relevance to the review topic. Only studies meeting a high standard of methodological rigor were included in the synthesis.

### Data extraction

Key data were extracted systematically from the included studies. Extracted information included:

• Study design and methodology.
• Participant demographics.
• Genetic factors examined.
• Key findings related to relationship stability and satisfaction.
• Interactions between genetic and environmental factors.

### Thematic synthesis

The extracted data were organized thematically to identify recurring patterns and key themes. Themes included genetic foundations of personality traits, attachment styles, emotional regulation, hormonal influences, sexual compatibility, communication styles, and mental health. The thematic synthesis approach allowed for an integrated understanding of how genetic factors contribute to relationship dynamics.

### Limitations and bias mitigation

To minimize bias, multiple reviewers independently screened and assessed the studies. Discrepancies were resolved through discussion and consensus. The use of multiple databases and diverse keywords reduced the risk of publication bias. However, the restriction to English-language studies may have excluded relevant research in other languages.

### PRISMA framework

**Fig. 1:**
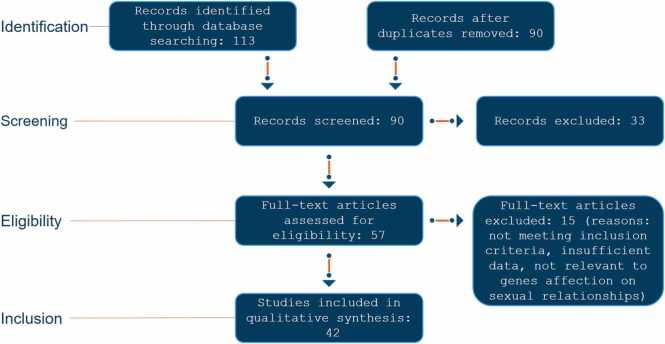
illustrates the PRIMSA flow diagram.

The study selection process was documented using the PRISMA (Preferred Reporting Items for Systematic Reviews and Meta-Analyses) guidelines. A PRISMA flow diagram (Fig. 1) illustrates the number of records identified, screened, and included in the review, along with reasons for exclusion at each stage.

## Genetic foundations of personality traits

Personality traits significantly shape the dynamics of sexual relationships, influencing how individuals interact with their partners, resolve conflicts, and maintain intimacy. The study of genetics has revealed that a considerable portion of individual differences in personality traits can be attributed to genetic factors. Twin and family studies have consistently shown that traits such as neuroticism, extraversion, and agreeableness have substantial heritability, underscoring the genetic basis of these characteristics [10].

Neuroticism, characterized by emotional instability and a tendency toward anxiety and depression, is one of the most influential personality traits affecting relationship dynamics. Individuals with high levels of neuroticism are prone to negative emotional states, which can lead to frequent conflicts and lower relationship satisfaction. Genetic studies have identified several loci associated with neuroticism, including variants in the gene encoding the serotonin transporter (5-HTTLPR), which plays a crucial role in regulating mood and emotional responses [11]. These genetic predispositions can exacerbate emotional reactivity and sensitivity to stress, often resulting in strained relationships and reduced stability.

Extraversion, on the other hand, is associated with sociability, assertiveness, and a higher propensity for positive emotions. This trait is generally linked to greater relationship satisfaction and stability, as extraverted individuals tend to engage more actively in social interactions and express affection more openly. Genetic research has identified associations between extraversion and polymorphisms in dopamine-related genes, such as the DRD4 gene, which influences the dopaminergic pathways related to reward processing and social behavior [12]. These genetic markers suggest that individuals with certain variants may be more predisposed to seek social interactions and maintain positive relational dynamics, thereby enhancing relationship satisfaction.

Agreeableness, characterized by traits such as trust, altruism, and cooperativeness, also plays a critical role in relationship dynamics. High levels of agreeableness are typically associated with greater relationship satisfaction, as these individuals are more likely to exhibit empathetic and supportive behaviors towards their partners. Genetic studies have pointed to the involvement of the oxytocin receptor gene (OXTR) in modulating agreeableness, highlighting the role of oxytocin in social bonding and pro-social behaviors [13]. Variations in the OXTR gene can influence the expression of these traits, affecting how individuals navigate interpersonal relationships and manage conflicts.

The interplay between these personality traits and genetic factors can create a complex matrix of influences that shape relationship dynamics. For instance, an individual with a genetic predisposition toward high neuroticism and low agreeableness may experience more frequent and intense conflicts in their relationship, leading to decreased satisfaction and stability. Conversely, someone with a genetic inclination toward high extraversion and agreeableness is likely to foster a more supportive and satisfying relationship environment.

Furthermore, genetic interactions between neurotransmitter systems are essential in personality expression. The balance between serotonin and dopamine activity is particularly relevant, as heightened neuroticism often coincides with increased serotonin transporter sensitivity, while extraversion correlates with greater dopamine receptor activity. These interactions provide a more comprehensive genetic framework for understanding personality-driven relationship behaviors.

## Attachment styles and genetic influences

Attachment styles, a central concept in understanding relationship dynamics, are influenced by both early environmental experiences and genetic factors. These styles, typically categorized as secure, anxious, and avoidant, significantly impact relationship stability and satisfaction. Secure attachment is characterized by comfort with intimacy and autonomy, while anxious attachment involves a preoccupation with relationships and fear of abandonment. Avoidant attachment, on the other hand, is marked by a discomfort with closeness and a preference for emotional distance. Research has increasingly focused on the genetic underpinnings of these attachment styles, shedding light on how inherited traits contribute to relational behaviors and outcomes [14].

Genetic studies have revealed that attachment styles have a heritable component, with estimates suggesting that approximately 30–50 % of the variance in attachment behaviors can be attributed to genetic factors. For instance, variations in the oxytocin receptor gene (OXTR) have been linked to differences in attachment behaviors. Oxytocin, often referred to as the "love hormone," plays a crucial role in social bonding and affiliative behaviors. Specific polymorphisms in the OXTR gene have been associated with secure attachment, promoting trust and emotional intimacy in relationships [15]. Individuals with these genetic variants are more likely to develop secure attachment styles, which in turn contribute to greater relationship stability and satisfaction.

However, focusing solely on OXTR overlooks the polygenic nature of attachment behaviors. Other relevant genes include AVPR1A, which encodes the vasopressin receptor and is implicated in social bonding, and SLC6A4, which regulates serotonin transport and influences stress response. These genes collectively shape an individual's attachment tendencies, with polygenic risk scores providing a more accurate measure of genetic predispositions to attachment styles.

Conversely, anxious attachment has been associated with genetic factors related to heightened sensitivity to stress and emotional dysregulation. Variants in genes involved in the hypothalamic-pituitary-adrenal (HPA) axis, such as the glucocorticoid receptor gene (NR3C1), have been implicated in anxious attachment. These genetic predispositions can lead to increased cortisol production in response to stress, exacerbating fears of abandonment and relational insecurity [16]. Consequently, individuals with these genetic traits may experience more frequent relationship conflicts and lower satisfaction due to their heightened emotional reactivity.

Avoidant attachment, characterized by a reluctance to depend on others and a preference for self-reliance, has also been linked to genetic factors. Studies have identified associations between avoidant attachment and polymorphisms in the dopamine receptor D2 gene (DRD2), which is involved in reward processing and social motivation. These genetic variations may reduce the rewarding aspects of social interactions, leading individuals to distance themselves emotionally from their partners [17]. This avoidance of intimacy can undermine relationship satisfaction and stability, as emotional closeness is a key component of fulfilling relationships.

The interplay between genetic predispositions and early environmental influences is critical in shaping attachment styles. Gene-environment interactions can either mitigate or exacerbate genetic tendencies toward particular attachment behaviors. For example, supportive and nurturing early caregiving can promote secure attachment even in individuals with genetic vulnerabilities to anxious or avoidant styles. Conversely, adverse childhood experiences can amplify genetic risks, leading to maladaptive attachment behaviors that hinder relationship success [18].

Developmental studies further reinforce the role of epigenetics in attachment formation. Research has demonstrated that early-life stress can induce DNA methylation changes in OXTR and NR3C1, altering gene expression patterns associated with social bonding and stress regulation. These findings highlight the dynamic nature of genetic and environmental interactions in shaping attachment behaviors across the lifespan.

## Genetic basis of emotional regulation

Emotional regulation is a critical factor in maintaining relationship satisfaction and stability. The ability to manage and express emotions appropriately plays a pivotal role in how individuals handle stress, resolve conflicts, and communicate with their partners. Recent research has identified several genes that influence emotional regulation, highlighting the genetic basis of these essential skills and their impact on relationships [19].

One of the key genes involved in emotional regulation is the serotonin transporter gene (SLC6A4), which affects the reuptake of serotonin, a neurotransmitter crucial for mood regulation. Variations in this gene, particularly the short allele of the 5-HTTLPR polymorphism, have been associated with increased sensitivity to emotional stimuli and greater emotional reactivity [20]. However, conflicting studies suggest that the impact of this polymorphism on emotional regulation is moderated by environmental influences, such as early childhood experiences and stress exposure. Some studies report no significant association between SLC6A4 and emotional reactivity, underscoring the complexity of gene-environment interactions in emotional regulation.

Another significant gene is the catechol-O-methyltransferase (COMT) gene, which is involved in the degradation of dopamine, a neurotransmitter associated with reward and pleasure. The COMT gene has a well-known polymorphism, Val158Met, where the Met allele is linked to lower enzymatic activity and higher levels of dopamine in the prefrontal cortex. This variation is associated with better executive function and emotional control [21]. Individuals with the Met allele typically exhibit better emotional regulation, which can enhance relationship satisfaction by promoting more adaptive responses to stress and facilitating effective conflict resolution.

The oxytocin receptor gene (OXTR) also plays a crucial role in emotional regulation and social bonding. Oxytocin, often called the "love hormone," influences trust, empathy, and social communication. Variants of the OXTR gene, such as rs53576, have been linked to differences in emotional sensitivity and stress reactivity [13]. DNA methylation of the OXTR gene has been associated with individual differences in emotional expression, highlighting the role of epigenetic mechanisms in regulating emotional behavior and relationship satisfaction.

Genetic variations can also affect the hypothalamic-pituitary-adrenal (HPA) axis, a central stress response system. Polymorphisms in the glucocorticoid receptor gene (NR3C1) can alter cortisol production and stress responses, impacting emotional regulation [16]. Individuals with certain NR3C1 variants may exhibit heightened stress responses, which can negatively affect their ability to manage emotions during conflicts and reduce relationship satisfaction. Conversely, those with genetic profiles that promote more balanced stress responses are likely to handle relational stressors more effectively, contributing to greater relationship stability.

The ability to regulate emotions effectively is crucial for maintaining positive relationship dynamics. Genetic predispositions that influence emotional regulation can either support or undermine relationship satisfaction and stability. For instance, individuals with genetic variants that promote better emotional control and stress management are more likely to navigate conflicts constructively and maintain harmonious relationships. In contrast, those with genetic predispositions towards emotional dysregulation may struggle with conflict resolution, leading to greater relationship dissatisfaction.

## Hormonal influences and genetic factors

Hormones play a crucial role in regulating bonding and intimacy in human relationships. Among these, oxytocin and vasopressin are particularly significant due to their involvement in social bonding, attachment, and emotional regulation. Genetic variations that influence the regulation and function of these hormones can profoundly impact relationship dynamics and success.

Oxytocin, often dubbed the "love hormone," is central to the formation and maintenance of social bonds. It facilitates trust, empathy, and emotional connection, all of which are essential components of intimate relationships. Genetic variations in the oxytocin receptor gene (OXTR) have been shown to affect how individuals experience and express these social behaviors. For instance, polymorphisms such as rs53576 in the OXTR gene are associated with differences in social cognition and stress reactivity [13]. Individuals with certain alleles of this gene may have a greater capacity for empathy and social bonding, leading to higher relationship satisfaction and stability. Conversely, those with variants linked to reduced oxytocin receptor sensitivity may struggle with emotional closeness, potentially undermining relationship success.

Vasopressin, another hormone closely related to oxytocin, also plays a pivotal role in social behaviors and pair bonding. Genetic studies have identified the vasopressin receptor 1a gene (AVPR1A) as a critical factor in modulating these effects. Variations in the AVPR1A gene are associated with differences in social bonding and affiliative behaviors. Research has demonstrated that specific polymorphisms in AVPR1A can influence partner bonding and attachment behaviors, with certain alleles linked to more robust social connections and greater relationship satisfaction [22]. These genetic influences highlight the biological underpinnings of relationship dynamics and the importance of hormonal regulation in fostering intimacy.

Beyond oxytocin and vasopressin, testosterone and estrogen also play essential roles in relationship dynamics. Testosterone has been linked to dominance behaviors, competitiveness, and mate-seeking tendencies. While higher testosterone levels can promote assertiveness and attraction, excessive dominance can sometimes lead to relationship instability. In contrast, estrogen has been associated with emotional bonding, sensitivity, and nurturing behaviors, promoting relationship closeness and caregiving tendencies. The balance between testosterone and estrogen, along with genetic variations in their respective receptors, can influence relationship satisfaction and stability.

Despite the growing body of research on hormonal influences, there are several limitations that must be acknowledged. Many studies rely on small sample sizes, making it difficult to generalize findings across diverse populations. Additionally, the effects of hormone-related genetic variants may be moderated by environmental and lifestyle factors, such as stress, diet, and social interactions. Furthermore, hormonal studies often face methodological challenges, such as accurately measuring hormone levels over time due to their fluctuations in response to external stimuli.

The interplay between genetic predispositions and hormonal responses extends to broader aspects of relationship success. For example, individuals with genetic profiles that promote efficient oxytocin and vasopressin signaling are generally better equipped to handle stress and engage in positive social interactions. This genetic advantage can lead to more effective communication, greater emotional support, and higher overall relationship satisfaction. Conversely, genetic variants that impair hormone signaling pathways may contribute to difficulties in forming and maintaining intimate bonds, increasing the risk of relationship dissatisfaction and instability.

Furthermore, the interaction between genetic predispositions to hormonal responses and environmental factors can significantly influence relationship outcomes. For instance, supportive and nurturing environments can enhance the positive effects of favorable genetic variants, promoting greater bonding and intimacy. On the other hand, stressful or adverse environments may exacerbate the challenges faced by individuals with less favorable genetic profiles, leading to poorer relationship outcomes [23].

## Sexual compatibility and genetic compatibility

Genetic compatibility plays a significant role in sexual attraction and relationship satisfaction. This concept posits that certain genetic combinations between partners can lead to higher levels of attraction and more satisfying relationships. One of the most studied genetic systems in this context is the major histocompatibility complex (MHC), a group of genes involved in immune system functioning. MHC genes are highly polymorphic, and research suggests that individuals are subconsciously attracted to partners with different MHC alleles, which may provide evolutionary advantages for offspring [24]. However, findings on MHC compatibility in humans remain inconsistent, with some studies failing to replicate the association between MHC dissimilarity and relationship satisfaction.

The theory of MHC-related sexual attraction is supported by the idea that diverse MHC combinations can enhance immune system robustness in offspring, thus increasing their chances of survival. Some studies have shown that MHC dissimilarity between partners correlates with higher levels of sexual attraction and satisfaction. For instance, Wedekind et al.'s seminal research demonstrated that women prefer the scent of men with dissimilar MHC genes, a preference that is thought to be mediated by olfactory cues [25]. However, other research has contradicted these findings, showing no significant preference for MHC-dissimilar partners.

Further research has explored how MHC compatibility influences long-term relationship satisfaction. Couples with greater MHC dissimilarity tend to report higher levels of relationship satisfaction and sexual fulfillment. This increased satisfaction is likely due to a combination of factors, including enhanced immune benefits for offspring and reduced likelihood of fertility issues. However, some studies indicate that the effects of MHC compatibility on relationship satisfaction may be minor or influenced by cultural and environmental factors [24].

The influence of MHC on partner selection has also been linked to pheromones, chemical signals believed to play a role in sexual attraction. Pheromonal communication is hypothesized to convey information about genetic compatibility, particularly MHC diversity. However, the empirical support for this hypothesis remains limited, with some studies failing to find a strong correlation between pheromonal attraction and genetic compatibility [26]. Given the mixed findings, the role of pheromones in human mate selection should be interpreted with caution.

Beyond MHC, other genetic markers also contribute to sexual compatibility and relationship satisfaction. For example, genetic variations in neurotransmitter systems, such as those involving dopamine and serotonin, can influence personality traits and sexual behaviors, thereby affecting relationship dynamics. Couples with compatible genetic profiles in these systems may experience more synchronized sexual desires and behaviors, leading to higher relationship satisfaction [17]. This genetic alignment can enhance emotional and sexual intimacy, which are critical components of a successful relationship.

## Genetic contributions to communication styles

Effective communication is a cornerstone of healthy relationships, significantly impacting relationship stability and satisfaction. Genetic factors play a crucial role in shaping communication patterns and styles within relationships. Variations in genes that influence neurotransmitter systems, such as those regulating dopamine and serotonin, contribute to individual differences in communication behaviors, including expressiveness, responsiveness, and conflict resolution.

One prominent area of research focuses on the serotonin transporter gene (SLC6A4) and its role in emotional regulation and communication. The SLC6A4 gene has a well-known polymorphism, 5-HTTLPR, with short (S) and long (L) alleles. Individuals with the S allele tend to exhibit heightened emotional sensitivity and reactivity, which can influence their communication styles. These individuals may be more prone to emotional expressiveness and may have greater difficulty regulating their emotions during conflicts, potentially leading to more volatile and less effective communication [19]. On the other hand, individuals with the L allele typically have better emotional regulation, contributing to more stable and constructive communication patterns.

The role of the oxytocin receptor gene (OXTR) in communication has also been extensively studied. Oxytocin, known for its role in social bonding and emotional regulation, significantly affects communication behaviors. Variations in the OXTR gene, such as the rs53576 polymorphism, are associated with differences in empathy, social sensitivity, and communicative responsiveness. Individuals with certain OXTR genotypes are more likely to exhibit higher levels of empathy and supportive communication, which can enhance relationship satisfaction and stability [13]. Conversely, those with less favorable genotypes may struggle with empathic communication, potentially leading to misunderstandings and conflicts within relationships.

The previously suggested role of the dopamine receptor D4 gene (DRD4) in communication remains speculative, with limited empirical support [17]. While some studies suggest that polymorphisms in DRD4 may contribute to novelty-seeking behaviors and impulsivity, the direct link between these genetic variants and communication styles in relationships is not well-established. Instead, gene-environment interactions appear to play a more significant role in shaping communication patterns. For example, individuals with genetic predispositions to communication difficulties may benefit from interventions such as relationship counseling and behavioral training, which can mitigate genetic risks and improve relational outcomes [23].

## Mental health, genetic predispositions, and relationship outcomes

Genetic predispositions to mental health conditions significantly influence relationship stability and satisfaction. Understanding how these genetic factors contribute to mental health challenges can shed light on the complexities of relationship dynamics and offer strategies for managing their impact.

Mental health conditions such as depression, anxiety, and bipolar disorder have well-established genetic components. Variations in genes related to neurotransmitter function, stress response, and neural plasticity can predispose individuals to these conditions. For instance, the serotonin transporter gene (SLC6A4) is linked to susceptibility to depression and anxiety. Individuals with the short allele of the 5-HTTLPR polymorphism are more vulnerable to these conditions due to less efficient serotonin reuptake, leading to prolonged stress responses and negative emotional states [19]. This genetic vulnerability can strain relationships by increasing the likelihood of emotional instability, conflict, and reduced emotional support.

Bipolar disorder, characterized by mood swings between depression and mania, is also influenced by genetic factors. Variants in the CACNA1C gene, which affects calcium channel function, have been associated with bipolar disorder. These genetic predispositions can lead to unpredictable mood changes, creating challenges in maintaining stable and satisfying relationships [17]. Partners of individuals with bipolar disorder may struggle with the inconsistency in emotional availability and the difficulties in managing the disorder’s impact on daily life.

Genetic predispositions to mental health conditions not only affect the individual but also influence the partner and the relationship as a whole. The stress associated with managing a partner’s mental health condition can lead to caregiver fatigue, reduced relationship satisfaction, and increased conflict. Moreover, the stigma and lack of understanding surrounding mental health issues can further exacerbate these challenges, making it harder for couples to seek and receive the support they need [13].

Despite these challenges, various psychiatric interventions can help mitigate the impact of genetic predispositions on relationships. Cognitive Behavioral Therapy (CBT) has been shown to improve emotional regulation in individuals with genetic vulnerabilities to mood disorders, reducing relational conflicts and enhancing communication. Additionally, couples therapy can provide structured support for navigating the complexities of mental health in relationships, promoting mutual understanding and effective coping strategies.

Furthermore, comorbid genetic risks—such as the overlap between anxiety and depression—can compound relationship difficulties. Individuals with multiple genetic predispositions to mental health conditions may experience greater relational stress, highlighting the importance of early intervention and ongoing support for couples managing these challenges [27], [28].

## Interplay between genetics and environment

The intricate interplay between genetics and environment plays a crucial role in shaping relationship outcomes. Gene-environment interactions refer to the dynamic process where genetic predispositions and environmental influences jointly affect individual behaviors and relational dynamics. This concept acknowledges that while genetic factors provide a foundation for certain traits and tendencies, environmental contexts significantly modulate these genetic influences, resulting in varied relationship outcomes.

One prominent example of gene-environment interaction is the role of the serotonin transporter gene (SLC6A4) in emotional regulation and relationship satisfaction. Individuals carrying the short allele of the 5-HTTLPR polymorphism in the SLC6A4 gene are more susceptible to stress and negative emotional states. However, supportive and nurturing environments can buffer these genetic vulnerabilities, leading to better emotional regulation and improved relationship satisfaction. Conversely, adverse environments, such as those characterized by high levels of stress or conflict, can exacerbate the negative effects of this genetic predisposition, resulting in poorer relationship outcomes [19].

Environmental factors can also interact with genetic predispositions to influence attachment styles and relationship stability. For instance, the oxytocin receptor gene (OXTR) is associated with social bonding and emotional regulation. Research indicates that individuals with certain OXTR genotypes are more responsive to environmental cues related to social support and attachment. In supportive environments, these individuals tend to develop secure attachment styles, characterized by trust, emotional intimacy, and relationship stability. However, in environments lacking social support or characterized by neglect, the same genetic predispositions can lead to insecure attachment styles, such as anxious or avoidant attachment, which negatively impact relationship satisfaction and stability [13].

Cultural and socioeconomic factors further influence gene-environment interactions. In collectivist cultures, strong social support systems may buffer genetic risks for attachment insecurity, fostering greater relationship stability. Conversely, in individualistic cultures where self-reliance is emphasized, genetic predispositions toward anxiety or avoidance in relationships may be exacerbated by weaker social safety nets. Similarly, socioeconomic status can shape the effects of genetic predispositions on relationships—individuals with genetic risks for emotional dysregulation may fare better in resource-rich environments where mental health support and relationship counseling are readily available, whereas those in lower socioeconomic settings may struggle with limited access to such resources.

The interplay between genetics and environment is further exemplified by the impact of childhood experiences on adult relationship outcomes. Childhood environments characterized by warmth, stability, and secure attachments can mitigate the adverse effects of genetic predispositions to mental health conditions and enhance relationship satisfaction in adulthood [29]. For example, individuals with genetic risk factors for anxiety or depression who experienced supportive parenting are less likely to exhibit these conditions in adulthood, leading to healthier and more stable relationships. Conversely, adverse childhood experiences, such as abuse or neglect, can amplify genetic risks and result in poorer mental health and relationship outcomes [30].

## Case studies and empirical evidence

Empirical evidence and case studies provide substantial insights into the genetic contributions to relationship stability and satisfaction. Various studies, including longitudinal and twin studies, have illuminated the complex interplay between genetic factors and relationship outcomes.

One of the seminal studies in this field is the Minnesota Twin Family Study, which has extensively investigated the genetic and environmental influences on psychological traits and behaviors, including relationship dynamics. This longitudinal study has followed twins from adolescence into adulthood, allowing researchers to disentangle the contributions of genetics and environment to various life outcomes. Findings from this study indicate that genetic factors account for a significant proportion of the variance in traits like emotional regulation, personality, and social behaviors, which are crucial for relationship stability and satisfaction [31].

A key finding from twin studies is the heritability of marital satisfaction. For instance, research conducted by Spotts et al. [32] utilized data from monozygotic and dizygotic twins to examine the genetic and environmental influences on marital satisfaction. The study found that approximately 30–40 % of the variance in marital satisfaction could be attributed to genetic factors, with the remainder influenced by unique environmental factors. This suggests that while genetic predispositions play a significant role, individual experiences and contexts are equally important in shaping marital satisfaction.

To supplement large-scale studies, qualitative case studies provide nuanced insights into real-life relationship dynamics. For example, a case study by O’Connell and Coombes [33] explored the relationship dynamics of couples where one partner had a genetic predisposition to bipolar disorder. The study highlighted how genetic factors interacted with environmental stressors to influence relationship stability and satisfaction. It also emphasized the importance of therapeutic interventions and support systems in mitigating the adverse effects of genetic predispositions on relationships.

To ensure methodological rigor, studies included in this review were selected based on criteria such as sample size, replication across populations, and assessment by established frameworks like the Critical Appraisal Skills Programme (CASP). This approach ensures that the studies analyzed provide robust and replicable insights into genetic contributions to relationship dynamics.

## Ethical and social implications

The exploration of genetic contributions to relationship stability and satisfaction raises significant ethical and social considerations. As genetic research continues to unveil the biological underpinnings of human behavior, it becomes imperative to address the ethical implications and potential societal impacts of such findings.

One primary ethical concern is privacy and the potential misuse of genetic information. Genetic data is highly sensitive, and there is a risk that it could be used to discriminate against individuals in various contexts, including employment, insurance, and personal relationships. The possibility of genetic screening for traits associated with relationship stability or satisfaction could lead to stigmatization and unjust treatment of individuals based on their genetic profiles [34]. Ensuring robust privacy protections and ethical guidelines for the use of genetic data is crucial to prevent misuse and discrimination.

Another ethical issue is the concept of genetic determinism, which suggests that genetic factors wholly dictate individual behaviors and relationship outcomes. This perspective can undermine the importance of environmental influences and personal agency in shaping relationships. Emphasizing genetic contributions without acknowledging the complex interplay with environmental factors risks oversimplifying human behavior and reducing individuals to their genetic predispositions [35]. Ethical communication of genetic research findings must balance the role of genetics with the recognition of personal choice and contextual influences.

Real-world ethical dilemmas further highlight these concerns. For instance, the use of genetic testing in dating applications raises questions about privacy, consent, and the potential commodification of genetic compatibility. While some argue that genetic insights could enhance relationship success, others warn that such practices might reinforce genetic determinism and exclude individuals based on perceived genetic "desirability" [36]. Policymakers and ethicists must consider these risks when evaluating the implications of genetic research in matchmaking services.

The ethical and social implications of genetic research in relationships necessitate a multidisciplinary approach involving geneticists, ethicists, sociologists, and policymakers. Collaborative efforts are required to develop ethical guidelines that protect individuals' rights, promote informed consent, and address the broader societal impacts of genetic findings. Public engagement and education are also critical to foster a nuanced understanding of genetic research and its implications for relationships [37].

## Future directions and research needs

The field of genetics and relationship science is burgeoning with opportunities for future research, aimed at deepening our understanding of how genetic factors influence relationship stability and satisfaction. Despite significant advancements, several research gaps and potential areas for future investigation remain.

One crucial area for future research is the exploration of gene-environment interactions in greater detail. While it is well-established that both genetic predispositions and environmental factors contribute to relationship outcomes, the specific mechanisms through which these interactions occur are not fully understood. Future studies should aim to elucidate how different environmental contexts, such as socioeconomic status, cultural background, and life stressors, interact with genetic factors to influence relationship dynamics [32]. Longitudinal research designs that track individuals and couples over extended periods can provide valuable insights into these complex interactions.

Additionally, there is a need for more research on the role of epigenetics in relationship science. Epigenetic mechanisms, which involve changes in gene expression without altering the underlying DNA sequence, are influenced by environmental factors and can have lasting effects on behavior and mental health. Investigating how epigenetic modifications impact relationship-related traits and behaviors can offer a more nuanced understanding of the biological underpinnings of relationship stability and satisfaction [13]. This line of research requires sophisticated methodologies, including advanced genomic sequencing and bioinformatics tools.

Future research should also focus on conducting large-scale genome-wide association studies (GWAS) to identify novel genetic markers associated with relationship dynamics. The implementation of polygenic risk scores (PRS) to predict relationship-related traits such as emotional regulation, attachment styles, and communication tendencies could provide more precise insights into genetic contributions to relationships [38]. Furthermore, increasing the diversity of study populations is essential to improve the generalizability of findings beyond Western, educated, industrialized, rich, and democratic (WEIRD) populations [39].

The study of epigenetics should be expanded to explore how early-life experiences shape genetic expression related to bonding and emotional regulation. Examining the neural correlates of relationship stability through neuroimaging techniques such as functional magnetic resonance imaging (fMRI) and positron emission tomography (PET) could further elucidate the biological basis of relationship dynamics [40]. Emerging technologies such as CRISPR-Cas9 and gene-editing tools may also contribute to future research by allowing scientists to investigate the genetic basis of social behaviors in controlled settings, although ethical considerations surrounding their application in human studies must be carefully addressed [41].

Artificial intelligence (AI) and machine learning are increasingly being used to predict relationship satisfaction based on genetic and behavioral data. AI-driven models can integrate genetic, psychological, and environmental variables to generate personalized relationship counseling strategies. Predictive analytics can help identify at-risk couples who may benefit from early interventions to improve relationship outcomes. The integration of AI with genetic research presents an opportunity to refine our understanding of how biological and environmental factors interact to shape relationship satisfaction and stability.

Finally, interdisciplinary research that integrates insights from psychology, sociology, genetics, and neuroscience will be crucial in addressing the multifaceted nature of relationships. Collaborative efforts can bridge the gaps between these fields, fostering a more comprehensive understanding of how genetic and environmental factors jointly shape relationship outcomes [42]. Such interdisciplinary approaches can also inform the development of holistic interventions and policies aimed at promoting healthy and satisfying relationships.

## Conclusion

In conclusion, this literature review highlights the significant influence of genetic factors on relationship stability and satisfaction, encompassing personality traits, attachment styles, emotional regulation, hormonal influences, and communication patterns. Findings indicate that while heritable traits like neuroticism and agreeableness play crucial roles, genetic influences do not operate in isolation but interact dynamically with environmental factors.

The evidence from longitudinal and twin studies underscores the complexity of genetic contributions to relationships, reinforcing that genetic predispositions shape but do not dictate relational outcomes. The integration of neuroimaging, epigenetics, and AI-driven predictive modeling presents promising directions for future research, offering deeper insights into the biological and environmental determinants of relationship dynamics.

Real-world applications of these findings can inform targeted interventions, such as personalized relationship counseling based on genetic predispositions, and public health initiatives designed to foster stable and satisfying relationships. However, ethical considerations remain paramount. The risk of genetic determinism must be critically evaluated, ensuring that research findings are communicated responsibly and that genetic data privacy is safeguarded.

Ultimately, while genetics provides valuable insights into relationship dynamics, environmental support systems, therapy, and personal agency remain integral in fostering healthy and fulfilling relationships. Future research should continue to explore the interplay between biology and environment to develop evidence-based interventions that enhance relationship stability and well-being.

## Ethics approval and consent to participate

Not applicable.

## Consent for publication

Not applicable.

## Funding

Not applicable.

## Declaration of Competing Interest

There is no potential competing interests***.***

## Data Availability

Data sharing not applicable to this article as no data-sets were generated or analyzed during the current study
